# The *Penicillium chrysogenum tom1* Gene a Major Target of Transcription Factor MAT1-1-1 Encodes a Nuclear Protein Involved in Sporulation

**DOI:** 10.3389/ffunb.2022.937023

**Published:** 2022-07-14

**Authors:** Barbara Ramšak, Ulrich Kück

**Affiliations:** Allgemeine und Molekulare Botanik, Ruhr-Universität Bochum, Fakultät für Biologie und Biotechnologie, Bochum, Germany

**Keywords:** Mating-type transcription factor, *Penicillium chrysogenum*, sporulation, fluorescence microscopy, yeast two-hybrid analysis, electrophoretic mobility shift assay

## Abstract

Fungal mating-type loci (*MAT*) encode transcription factors (TFs) MAT1-1-1 and MAT1-2-1, which govern sexual reproduction as well as other developmental processes. In *Penicillium chrysogenum*, the major producer of the beta-lactam antibiotic penicillin, a recent chromatin immunoprecipitation followed by sequencing (ChIP-seq) analysis identified 254 genes as direct targets of MAT1-1-1, many of which encode thus far uncharacterized proteins. Here, we characterized one of the major targets of MAT1-1-1, the *tom1* gene, which encodes a protein highly conserved within the group of Eurotiomycetes fungi. Using fluorescence microscopy, we demonstrated binding of MAT1-1-1 to the *tom1* promoter by reporter gene analysis. Extensive electrophoretic mobility shift assays (EMSAs) further showed that the promoter sequence of *tom1* is bound *in vitro* by both MAT1-1-1 and MAT1-2-1. This indicated an interaction between the two TFs, which was verified by yeast two-hybrid analysis. The sequence of *tom1* carries a nuclear localization sequence, and indeed its nuclear localization was verified by fluorescence microscopy. The *in vivo* function of *tom1* was investigated using *tom1* deletion strains, as well as a complementing strain where the wild-type *tom1* gene was reintroduced. We found a clear sporulation defect in the deletion strain, which became more evident when the fungi were grown at an elevated temperature of 31°C.

## Introduction

Fungal mating-type (*MAT*) loci determine the cell type specificity of haploid cells as well as the fate of the diploid zygotes, and thus control sexual development. In most ascomycetous fungi, the *MAT* locus is represented by a rather small genomic region ranging from 1 to 6 kb (Coppin et al., 1997; Kronstad and Staben, 1997; Dyer and Kück, 2017). The simplest *MAT* loci are commonly found among the members of the Eurotiomycetes (Ascomycota, Pezizomycotina) (Dyer et al., 2016). For example, the majority of self-sterile (heterothallic) *Penicillium* species employ bipolar reproduction systems, where *MAT1-1* and *MAT1-2* loci each encode only a single gene (Hoff et al., 2008; Ropars et al., 2012; Mahmoudjanlou et al., 2020). As a rule, the *MAT1-1* locus carries an alpha1 (α1-) domain transcription factor (TF) gene *(MAT1-1-1)*, whereas *MAT1-2* codes for a high mobility group (HMG) domain TF (*MAT1-2-1)* (Dyer and Kück, 2017).

Based on previous studies, besides sexual reproduction, MAT TFs have been implicated in other processes, such as hyphal morphology, asexual sporulation, and secondary metabolism (Zheng et al., 2013; Yu et al., 2018; Yong et al., 2020). Although the versatility of the MAT TFs has been demonstrated for numerous filamentous fungi, only a few reports considered their direct gene targets (Philley and Staben, 1994; Ait Benkhali et al., 2013; Becker et al., 2015).

*Penicillium chrysogenum* (syn. *Penicillium rubens*) is a major producer of the β-lactam antibiotic penicillin, and it is therefore considered to be one of the most valuable fungi in industrial microbiology (Elander, 2003; Kück et al., 2014). The *MAT* genes of *P. chrysogenum* have been intensively studied at the functional and molecular level (Hoff et al., 2008; Böhm et al., 2013; Böhm et al., 2015; Becker et al., 2015). We recently performed a chromatin immunoprecipitation followed by DNA sequencing (ChIP-seq) analysis, providing a comprehensive view of MAT1-1-1-driven transcriptional regulation (Becker et al., 2015). A core DNA-binding consensus motif for the *P. chrysogenum* MAT1-1-1 was found in 254 genes. Indeed, a portion of the MAT1-1-1 targets was represented by genes essential for mating recognition, e.g., pheromone receptor *pre1* and pheromone precursor *ppg1* (Hoff et al., 2008), as well as genes for *kex1* and *kex2* proteases, which are involved in processing pheromone precursors (Thomas et al., 1990). Moreover, the DNA-binding motif for MAT1-1-1 was also enriched in promoters of uncharacterized genes (Becker et al., 2015). Therefore, to fully clarify this broad regulatory role of MAT1-1-1, it is necessary to characterize target genes, whose functions are unknown.

Intriguingly, the statistically highest peak value (enrichment) identified by ChIP-seq analysis (Becker et al., 2015) was for an uncharacterized gene *Pc20g00090*, suggesting this gene receives a direct transcriptional input from MAT1-1-1. Similar values were found in subsequent ChIP-seq analysis when the *MAT1-1-1* gene was expressed under its native promoter (Kück and Krevet, unpublished data). *Pc20g00090* has recently been called ‘target of MAT1-1-1’, abbreviated as *tom1* (Ramsak et al., 2021).

Orthologues of *tom1* seem to be present in all members of the Eurotiomycetes, including the genera *Aspergillus* and *Penicillium*. Obviously, this is a conserved but so far uncharacterized gene for which insights into its regulation and function are highly desirable. Here, we greatly extended our *in vivo* binding assays of MAT1-1-1 to the *tom1* gene promoter. Furthermore, we determined the subcellular localization of the Tom1 protein in *P. chrysogenum* and demonstrated that a *tom1* deletion mutant is impaired in asexual sporulation at elevated temperatures. Based on DNA-binding studies and yeast two-hybrid analysis we propose that a heterodimerization between MAT1-1-1 and MAT1-2-1 could play a role in the regulation of *tom1* gene expression.

## Materials and Methods

### Strains, Media, and Growth Conditions

All microbial strains used in this study are listed in **Supplementary Table 1**. Recombinant plasmid construction and propagation were conducted in *Escherichia coli* strain *XL1* Blue MRF’, grown in Lysogeny Broth (LB) medium supplemented with appropriate antibiotics (Bullock et al., 1987; Sambrook and Russell, 2001). *Saccharomyces cerevisiae* strains PJ69-4a and PJ69-4α were propagated according to standard protocols (Sambrook and Russell, 2001). All *P. chrysogenum* strains were cultured on solid Complete Culture Medium (CCM) (Walz and Kück, 1991) for 6-7 days at 27°C in continuous light. *P. chrysogenum* transformants were selected on CCMS (CCM supplemented with 20% glucose) medium supplemented with 200 μg ml^-1^ nourseothricin or 40 μg ml^-1^ of phleomycin.

### Preparation of Nucleic Acids and cDNA Synthesis

Extraction of nucleic acids for the analysis of *P. chrysogenum* transgenic strains was done as described before (Hoff et al., 2010; Böhm et al., 2013). DNA-mediated transformation of *P. chrysogenum* was performed with linearized plasmids as described previously (Hoff et al., 2005; Kamerewerd et al., 2011). cDNA synthesis of *P. chrysogenum* for getting intron-free *MAT* genes to clone them into yeast two-hybrid- and pGEX-4T1 plasmids was carried out as described previously (Hoff et al., 2010; Böhm et al., 2013). The sequences for all primers used in this study are listed in the **Supplementary Table 2**.

### *In Vivo DsRed* Reporter Gene Analysis

For our *in vivo DsRed* expression analysis, a 965 bp upstream sequence directly adjacent to the translation initiation site of the *tom1* gene was analysed for the occurrence of any additional MAT1-1-1 binding sites using a Find Individual Motif Occurrences (FIMO) tool (Grant et al., 2011). Then, the 965 bp DNA fragment corresponding to the promoter of the *tom1* gene was amplified from *P. chrysogenum* genomic DNA with primers Pc20g00090_dsRed_ApaI_f and Pc20g00090_dsRed_HindIII. The amplified PCR product represented promoter variant P*tom1*_(0-965 bp),_ which was double digested with ApaI-HindIII, and subsequently ligated into the plasmid pDsRed digested with the same enzyme pair. The plasmid generated was named pDsRed_Ptom1. Additionally, two plasmids were constructed carrying different variants, P*tom1*_(-149-965 bp)_ and P*tom1*_(-222-965 bp)_, of the 3’-end-truncated *tom1* promoters. They were amplified with primer Pc20g00090_dsRed_ApaI_f paired with either Pc20g00090_826_ds Red_HindIII or Pc20g00090_753_ds Red_HindIII for P*tom1*_(-149-965 bp)_ and P*tom1*_(-222-965 bp)_, respectively. The resulting PCR products were inserted into pDsRed as described for the variant P*tom1*_(0-965 bp)_, generating plasmids pDsRed_Ptom1(-149) and pDsRed_Ptom1(-222). The constructed plasmids allowed expression of the *Discosoma* sp. red fluorescent gene *DsRed* under the control of the corresponding P*tom1* variants. The plasmids were ectopically integrated into the P2niaD18 strain expressing *egfp-MAT1-1-1*, which was generated previously and is called MAT1-ChIP (Becker et al., 2015). Subsequently, transformants were selected on phleomycin, and expression of the *DsRed* gene was confirmed by fluorescence microscopy. For each *tom1* promoter variant at least five positive transformants were screened. The fluorescence microscopy was done as previously described (Becker et al., 2015). All primers and plasmids used in this study are listed in **Supplementary Tables 2** and **3**.

### Synthesis and Purification of the MAT TFs

Plasmids pGEX-MAT1-1-1 or pGEX-MAT1-2-1 were used for heterologous expression of *MAT1-1-1* and *MAT1-2-1* genes. In these cases, both *MAT* genes were N-terminally fused to the glutathione-*S*-transferase (GST) tag from *Schistosoma japonicum*. Plasmid pGEX-MAT1-1-1 was described previously (Becker et al., 2015). For construction of the plasmid pGEX-MAT1-2-1, the *MAT1-2-1* gene sequence was amplified from cDNA of *P. chrysogenum* PC3 strain, with primers BspHI-MAT1-2-1_f and MAT1-2-1-EcoRI_r to cover the full-length coding sequence of the open reading frame. The 930 bp PCR product was then cloned into the BspHI/EcoRI linearized expression vector pGEX-4T1, resulting in plasmid pGEX-MAT1-2-1. The primers and plasmids are given in the Supplementary Material (**Supplementary Tables 2** and **3**).

For protein synthesis, plasmids pGEX-MAT1-1-1 and pGEX-MAT1-2-1 were transformed into *E. coli* BL21-Gold (DE3) pLysS cells (**Supplementary Table 1**). The synthesized GST-tagged recombinant proteins in the supernatant were purified using affinity chromatography as described previously (Ramsak et al., 2021). To obtain a tag free MAT1-2-1 protein, a 26 kDa GST was removed from GST-MAT1-2-1 by digestion overnight at 4°C, using Turbo -Tobacco etch virus- (TEV) Protease (MoBiTec GmBH, Göttingen, Germany) in buffer 150 mM NaCl, 27 mM KCl, 10 mM Na_2_HPO_4_, 18 mM KH_2_PO_4_, 2 mM DTT, pH 7.3. The released pure GST was captured using Glutathione Sepharose 4B resin (GE Healthcare). The flow-through containing pure MAT1-2-1 was collected and concentrated, resulting in a protein concentration of approximately 1 mg ml^-1^. Protein concentrations of the MAT TFs were determined using the Bradford protein assay. Purified proteins were fractionated on 12% (w/v) SDS-PAGE, followed by Coomassie Blue Staining (**Supplementary Figure 2**).

### DNA-Binding Assays

DNA binding of the MAT proteins was analysed by electrophoretic mobility shift assays (EMSAs) following the protocol described by Ramsak et al. (2021). Briefly, a fixed amount of 29 bp ^32^P-labeled double-stranded synthetic dsDNA probe, tom1-2, was mixed with purified recombinant GST-tagged MAT proteins and incubated in a 20 μl reaction mixture for 20 min at room temperature. For competition EMSAs experiments, varying concentrations of a specific or unspecific unlabelled synthetic dsDNA probe (tom1-2 or kex2-3) were used. The sequences for the tom1-2 and kex2-3 can be found in **Supplementary Table 2**. The kex2-3 probe was used as unspecific competitor, because it does not bind MAT1-1-1, as demonstrated previously (Becker et al., 2015). Samples were then resolved on native 4% polyacrylamide gels and electrophoresed for 2-3 h at 120 V. Gels were dried and subjected to standard autoradiographic procedures.

### Yeast Two-Hybrid Analysis

For yeast two-hybrid analysis (James et al., 1996) *MAT1-1-1* and *MAT1-2-1* fragments were amplified *via* PCR from cDNA of *P. chrysogenum* P2niaD18 (*MAT1-1*) and PC3 (*MAT1-2*) strains. We did not use the complete MAT1-1-1 sequence, since the full-length protein shows trans-activation activity in yeast cells. The amplified fragments were used to generate in-frame fusions with either the Gal4 DNA-binding domain (BD) or Gal4 activation domain (AD) encoded by bait pGBKT7 and pray pGADT7 plasmids, respectively. The plasmids were generated by classical restriction digestion and ligation-mediated cloning and were verified by sequencing. The resulting pGADT7 and pGBKT7 derivatives (**Supplementary Table 3**) were transformed into *S. cerevisiae* strains PJ69-4a and PJ69-4α, respectively. A full lists of the primers and plasmids used in this study are listed in the **Supplementary Tables 2** and **3**. Mating of yeast strains was carried out as described previously (Bloemendal et al., 2012; Kopke et al., 2013), and diploids containing both pray and bait plasmids were selected on the synthetic defined medium SD lacking both, leucine and tryptophan (SD-Leu-Trp). The functionality of the pGBKT7 derivates was tested with the plasmid pA-RanBPM (Tucker et al., 2009). Subsequently, screening for positive interactions was carried out on SD medium lacking histidine, leucine, tryptophan, and adenine (SD-Leu-Trp-His-Ade). Positive diploids were investigated for the expression of *lacZ* reporter gene, by transferring them on ‘SD-Leu-Trp-His-Ade’ plates containing X-α-Gal. Yeast transformants expressing α-galactosidase in response to a positive two-hybrid interaction turned blue.

### Construction of Deletion, Complementation, and Overexpression Strains

Plasmid KO-EN45_061320 was constructed for deletion of the *tom1* gene. The ~1kb 5′- and 3′-flanking sequences were amplified from *P. chrysogenum* genomic DNA with primers 5’-Flanke Pc20g00090_IF_f and 5’-Flanke Pc20g00090_IF_r; the 3′-flanking region was amplified with primers 3’-Flanke Pc20g00090_IF_f and 3’-Flanke Pc20g00090_IF_r. Subsequently, the amplified 5′- and 3′-flanking sequences were cloned into pD-NAT1 plasmid using the In-Fusion HD Cloning Kit (Takara Bio, Saint-Germain-en-Laye, France). The resulting KO-EN45_061320 plasmid carrying a nourseothricin (*nat1*) resistance gene was used for homologous integration into ΔPc*ku70* (Kopke et al., 2010). The latter lacks any resistance marker. The genotype of the Δ*tom1* deletion mutant was further analysed by PCR and Southern hybridization as described earlier (Hoff et al., 2005). A 575 bp 5´flank promoter sequence of *tom1* was used as a specific probe for Southern hybridization (**Supplementary Figure 1**).

Plasmid pGFP-Tom1_ble was generated for rescue of the Δ*tom1* deletion mutant and to investigate the subcellular localization of the *tom1*. The 844 bp *tom1* gene was amplified from genomic DNA with specific primers tom1_KpnI_f and tom1_EcoRI_r, which were extended by the recognition sites for KpnI and EcoRI, respectively. Subsequently, the amplified fragment was inserted into the KpnI-EcoRI linearized plasmid Yas1_ptrpC-phleo-egfp, resulting in plasmid pGFP-Tom1_ble. The latter carries the *egfp-tom1* fusion under the control of the *gpd* promoter and *trpC* terminator from *Aspergillus nidulans*. Plasmid pGFP-Tom1_ble was ectopically integrated into recipient strains Δ*tom1* and P2niaD18, resulting in complementation (*Δ*tom1::EGFP-Tom1) and overexpression (P2niaD18::EGFP-Tom1) strains.

### Light and Fluorescence Microscopy

Microscopic investigation of *P. chrysogenum* strains was performed as described (Becker et al., 2015; Schmidt et al., 2020). For subcellular localization of EGFP-Tom1, strains were inoculated on sterile CCM-covered slides and incubated for 24 h (vegetative growth) and 48 h (conidia formation) at 27°C. Nuclear DNA was stained with 5 mg ml^-1^ of 4,6-diamidino-2-phenylindole (DAPI) diluted in 0.7% (w/v) NaCl. Fluorescence microscopy was performed as previously described (Becker et al., 2015). Recorded images were processed with MetaMorph and Image J (Schneider et al., 2012).

### Sporulation Assay

To investigate sporulation, the reference, deletion, and complementation strains were grown on solid M322 medium (Wolfers et al., 2015) for 7 days at 27°C under continuous light. Spores were then harvested in physiological saline solution of 0.85% (w/v) NaCl. To quantify the spore production, 200 µl of 10^7^ spores ml^-1^ were spread on solid CCM medium and cultured for 7 days at 27°C or 31°C under continuous light or dark conditions. A circular disc of 1 cm diameter was cut out of the plate and dissolved in 10 ml buffer [0.85% NaCl (w/v), 0.01% (w/v) Tween] by boiling for 30 min. Next, each sample was treated with a sonifier (Sonifier 250; Brandson) for 6 min. Concentrations of spores were determined by light microscopy using an Abbe-Zeiss counting cell chamber. The measurements for each strain were performed in triplicate.

## Results

### *tom1* is an *in vivo* Target Gene of MAT1-1-1

**Figure 1:**
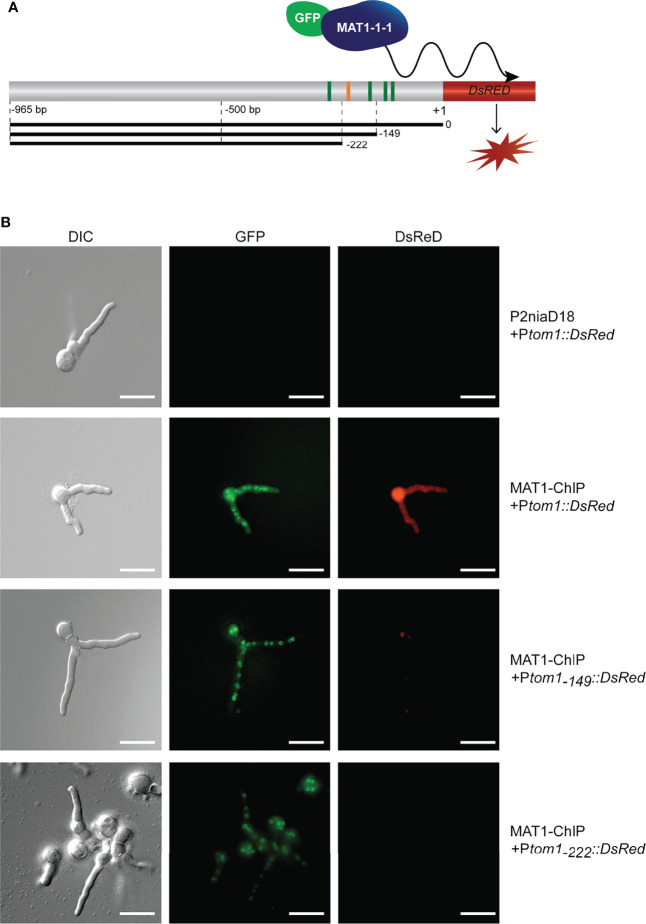
Identification of *tom1* as an *in vivo* target of MAT1-1-1 **(A)** Schematic representation of the *tom1* gene promoter region fused to the *DsRed* reporter gene. The consensus motif that binds MAT1-1-1 *in vitro* is indicated by orange bar, while other putative MAT1-1-1 motifs (p<0.001) identified by FIMO (Grant et al., 2011) are shown as green bars. The full-length P*tom1*_(0-965 bp)_ and truncated variants P*tom1*_(-149-965 bp)_ and P*tom1*_(-222-965 bp)_ are given as black bars. **(B)** *In vivo* association of MAT1-1-1 with the *tom1* promoter variants. Note, the *egfp-MAT1-1-1* is expressed (green fluorescence) by recipient MAT1-ChIP strain. Red fluorescence indicates the expression of the *DsRed* gene under the control of the *tom1* promoter. Scale bars, 20 μm.

Previous studies have shown that MAT1-1-1 binds *in vitro* to a consensus motif ‘CTATTGAG’ within the *tom1* gene promoter (Becker et al., 2015). To verify this binding *in vivo*, we performed a fluorescence study using *DsRed* reporter gene constructs under the control of different *tom1* promoter derivatives (**Figure 1**). The analysis of binding sites revealed a total of 5 hits (p<0.001), including the site identified in ChIP-seq analysis (Becker et al., 2015), adjacent to the translation initiation site (TIS) (**Figure 1A**). An intense red fluorescence signal was detected for transformants carrying variant P*tom1*_(0-965 bp)_ (**Figure 1B**), indicating that MAT1-1-1 indeed binds to the *tom1* promoter *in vivo*.

We extended our analysis by using two truncated variants (P*tom1*_(-149-965 bp),_ and P*tom1*_(-222-965 bp)_) of the *tom1* promoter. No red fluorescence was detected in any of these transformants, although P*tom1*_(-149-965 bp)_ still carries the verified consensus motif of MAT1-1-1 (**Figure 1A**). This indicates that the previously identified functional motif of MAT1-1-1 alone is not sufficient to activate transcription of the *tom1* gene *in vivo*. Overall, our results showed that the promoter of *tom1* is directly targeted by MAT1-1-1 *in vivo* in *P. chrysogenum*.

### The Tom1-2 Sequence is Bound by MAT1-1-1 and MAT1-2-1 in a Sequence-Specific Manner

**Figure 2:**
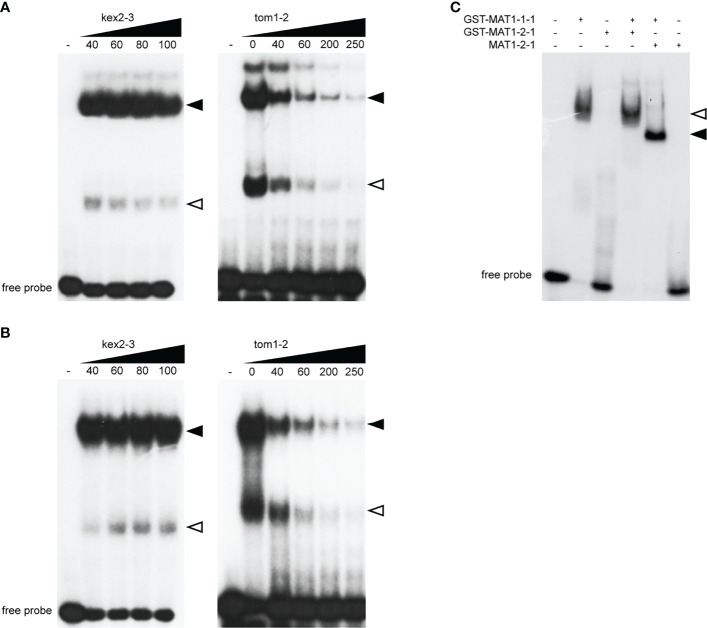
EMSAs show sequence-specific binding of the MAT1-1-1 and MAT1-2-1 proteins to the tom1-2. **(A)** EMSA image showing *in vitro* binding of GST-MAT1-1-1 to tom1-2 probe in presence of increasing competitor, nonspecific kex2-3 (left) and specific tom1-2 (right). Equal molar amounts (2.5 µM) of protein was mixed with unlabled competitor DNA in pmol, as indicated. **(B)** EMSA image showing *in vitro* binding of GST-MAT1-2-1 to tom1-2 probe in presence of nonspecific kex2-3 (left) and specific tom1-2 (right) competitor probe. Equal molar amounts (7 µM) protein was mixed with unlabled competitor DNA in pmol, as indicated. In **(A, B)** homodimers and heterodimers of GST-MAT1-1-1 in complex with tom1-2 are indicated by open and black arrowheads respectively. **(C)** EMSA showing cooperative binding of MAT1-1-1 and MAT1-2-1 to the tom1-2 derived from the promoter of the *tom1* gene. Equal molar amounts (4 µM) of each protein were co-incubated with tom1-2 and applied to the gel. The arrowheads indicate formation of GST-MAT1-1-1/GST-MAT1-2-1/tom1-2 (open arrowhead) and GST-MAT1-1-1/MAT1-2-1/tom1-2 (black arrowhead) complexes.

We previously demonstrated that a DNA probe tom1-2 derived from the promoter region of the *tom1* gene is efficiently bound by MAT1-1-1 *in vitro* (Becker et al., 2015). The consensus motif for MAT1-1-1 is centrally positioned in this probe. To further characterize the molecular role of MAT1-1-1 in regulating *tom1*, we performed electrophoretic shift mobility assays (EMSAs) (**Figure 2**). First, the GST-MAT1-1-1 protein was incubated with the native tom1-2 probe in the presence of increasing concentrations of unlabelled nonspecific competitor kex2-3 probe. Here, we observed no reduction in signal intensity of the shifted bands, indicating that the formation of the protein-tom1-2 DNA complex was not altered (**Figure 2A**). In contrast, when GST-MAT1-1-1 was incubated with its native specific competitor, signal intensity was significantly reduced (**Figure 2A**). These results suggest that MAT1-1-1 binds specifically to tom1-2.

In addition, our EMSA experiments also revealed that MAT1-2-1 binds the tom1-2 probe (**Figure 2B**). Although higher amounts of GST-MAT1-2-1 than GST-MAT1-1-1 protein were required for DNA binding, the binding specificity of MAT1-2-1 was the same, as confirmed by using the same specific and nonspecific (**Figure 2B**) competitor probes as for MAT1-1-1.

To further explore the DNA binding activity of GST-MAT1-1-1 and GST-MAT1-2-1, we performed additional EMSAs where both proteins were simultaneously incubated with the tom1-2 probe (**Figure 2C**). To exclude a possible GST tag contribution to binding, the 26 kDa GST tag was also removed from MAT1-2-1 to avoid interfering of GST in protein-protein interactions (**Supplementary Figure 2**). A slower migrating band was observed when GST-MAT1-1-1 was incubated with GST-MAT1-2-1 (**Figure 2C**). However, in the case of MAT1-2-1 without the GST tag, a band is observed migrating faster than GST-MAT1-2-1. This difference in migrating bands indicates that MAT1-2-1 takes part in the protein-DNA complex together with GST-MAT1-1-1. We used a lower amount of the MAT1-2-1 protein in **Figure 2C** than in **Figure 2B**, which explains the lack of binding, when MAT1-1-1 is absent in the samples.

### MAT1-1-1 Interacts With MAT1-2-1

**Figure 3:**
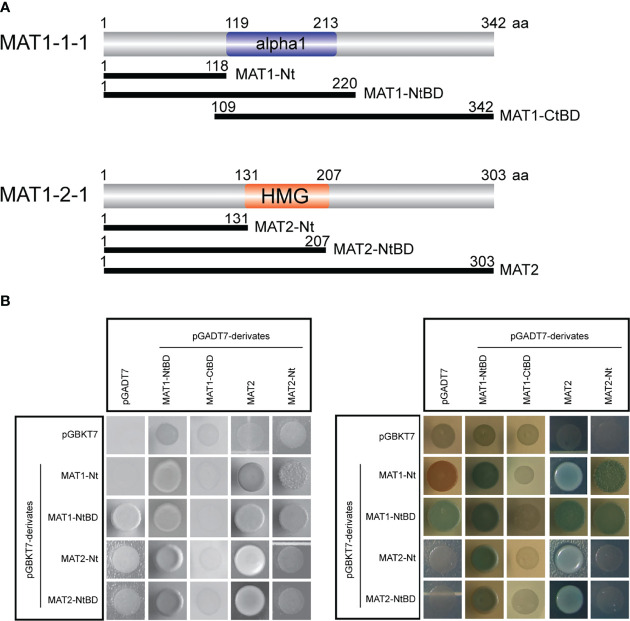
Proteins MAT1-1-1 and MAT1-2-1 physically interact. **(A)** Schematic representation showing truncated fragments of MAT1-1-1 and MAT1-2-1 TFs. The truncated fragments were fused to Gal4 AD (pGADT7 derivatives; MAT1-NtBD, MAT1-CtBD, MAT2, MAT2-Nt) and to Gal4 BD (pGBKT7 derivatives; MAT1-Nt, MAT1-NtBD, MAT2-Nt, MAT2-NtBD). Gal4 AD, Gal4 activation domain; Gal4 BD, Gal4 binding domain. **(B)** Yeast two-hybrid analysis of MAT proteins shows that MAT1-1-1 and MAT1-2-1 form homo- and heterodimer complexes. Yeast transformants expressing the Gal4 AD and Gal4 AD fusion constructs were selected on SD-Leu-Trp-His-Ade medium (left panel in black and white). The right panel shows α-galactosidase activity of yeast transformants selected on SD-Leu-Trp-His-Ade medium containing X-α-Gal. Blue color indicates positive protein interactions. Yeast transformants carrying empty plasmids pGADT7 and pGBKT7 were used as a negative control.

Since EMSA demonstrated that both MAT1-1-1 and MAT1-2-1 bind to the tom1-2 sequence (**Figure 2**), this suggests that the proteins interact with one another. To verify this hypothesis, we carried out yeast two-hybrid analysis with full-length and truncated protein fragments of MAT1-1-1 and MAT1-2-1 (**Figure 3A**). In addition, to screen for possible homodimeric protein interactions, we used MAT TFs fragments in both pGADT7 and pGBKT7- derivatives (**Figure 3A, B****)**. All yeast transformants were screened for growth on SD-Leu-Trp-His-Ade medium (**Figure 3A**), as well as for activity of the *lacZ* reporter gene (**Figure 3B**). As further control of a functional yeast interaction system, pA-RanBPM (Tucker et al., 2009) was used as a positive control (data not shown).

We found heterodimer formation with the following constructs. The N-terminus of MAT1-1-1 (1-118 aa; MAT1-Nt) interacts with both full-length MAT1-2-1 (1-303 aa; MAT2), and N-terminus of MAT1-2-1 (1-131 aa; MAT2-Nt) (**Figure 3A**). This result indicated that the N-termini of MAT TFs are sufficient to form heterodimeric complex. Further, we found several homodimer formations, e.g., between MAT1-Nt and fragment spanning N-terminus and DNA-binding domain of MAT1-1-1 (1-220 aa; MAT1-NtBD); and MAT2 with MAT2-Nt (**Figure 3A**). All these interactions were confirmed by activation of the *lacZ* reporter gene expression data (**Figure 3B**). In contrast, MAT1-1-1 polypeptide comprising only the DNA-binding domain and the C-terminus (109-342 aa; MAT1-CtBD) does not interact with any of the MAT protein fragments. From this data we conclude that the N-terminal part of MAT1-1-1 is necessary for the interaction with MAT1-2-1 or with itself (**Figure 3A, B****)**.

### Fluorescence Microscopy Demonstrates Localization of Tom1 Protein in the Nucleus

**Figure 4:**
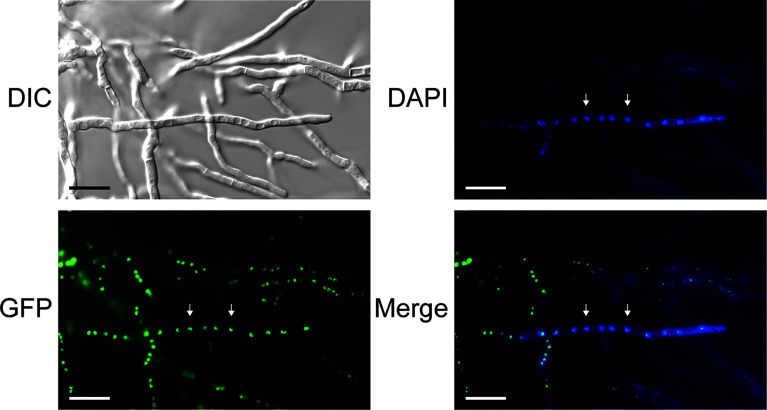
Differential interference contrast (DIC) and fluorescence microscopy to localize EGFP-Tom1 in subcellular structures. P2niaD18::EGFP-Tom1 transformants show nuclear localization of EGFP-Tom1 in mycelial cells. Arrowheads point to nuclei, which were identified by staining with 4′,6-diamidino-2-phenylindole (DAPI). Merge: merged images of DAPI and GFP fluorescence. Scale bars, 20 μm.

We previously noted that all *tom1* orthologues exhibit a putative C-terminal nuclear localization signal (NLS) (Ramsak et al., 2021). We further defined a short N-terminal linear proline-proline-x–tyrosine (PPxY, where x is any amino acid) motif of 42 to 45 residues (**Supplementary Figure 3**). Both the NLS and the PPxY motif are present in all Tom1 orthologues. To verify that the putative NLS directs Tom1 to the nucleus, plasmid pGFP-Tom1_ble containing an *egfp-tom1* fusion was constructed for cellular localization studies. A strong green fluorescence signal in the nuclei of hyphal cells was clearly observed for the P2niaD18*::egfp-tom1* transformants (**Figure 4**), indicating that *tom1* localizes to the nuclei of *P. chrysogenum*. In contrast, P2niaD18*::egfp* transformants expressing only the *egfp* gene showed an evenly distributed fluorescence in the cytoplasm (**Supplementary Figure 4**). During our investigation of transgenic sporulation strains, we observed an intense green fluorescence signal in mono- and binucleate conidia (**Supplementary Figure 4**). This result suggested that Tom1 plays a functional role in early vegetative development.

### *tom1* is Involved in Conidiation of *P. chrysogenum*

To clarify a possible role of Tom1 during vegetative growth, we generated Δ*tom1* mutants by replacing the *tom1* gene with a *nat1* resistance cassette using homologous recombination (**Supplementary Figure 1**). As a further control, the Δ*tom1* mutant was complemented with the *egfp*-*tom1* fusion, resulting in Δ*tom1*::*egfp-tom1* (**Supplementary Figure 4**). The strains were then further characterized for their sporulation properties and Δ*ku70*, Δ*tom1* and Δ*tom1*::*egfp-tom1* were grown on CCM medium for 7 d at 27°C and 31°C under constant light and dark conditions. 27°C is the optimal growth temperature for *P. chrysogenum*, although it does tolerate an increased temperature of 31°C; at yet higher temperatures growth is dramatically retarded.

**Figure 5:**
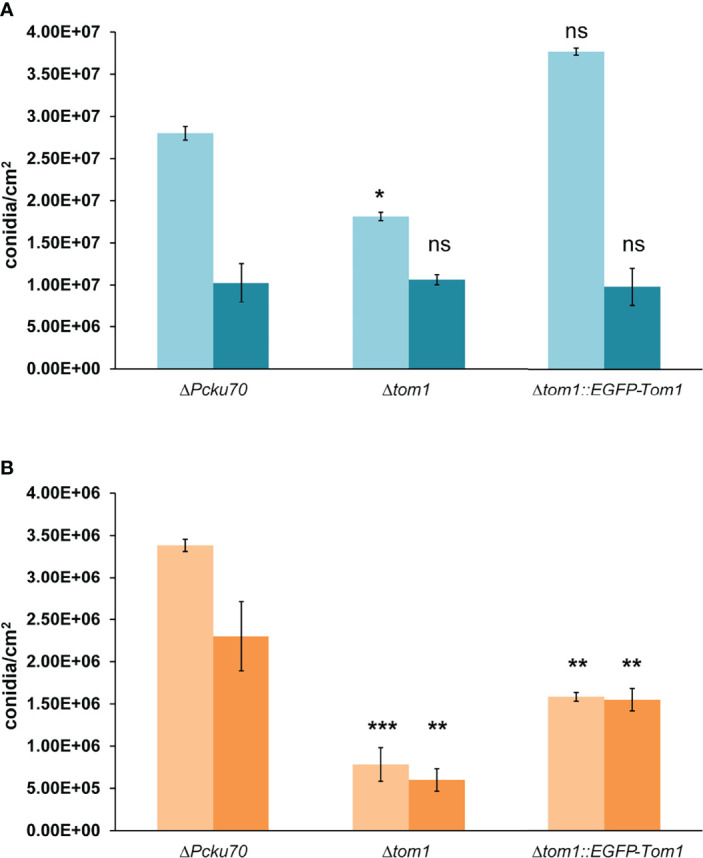
Quantitative assay of conidiation in Δ*tom1*. Quantification of conidia formation in the recipient ΔPc*ku70*, the deletion Δ*tom1* and corresponding complementation Δ*tom1::EGFP-Tom1* strains. Strains were grown on solid CCM for 168 h exposed to either constant light or constant dark at 27°C **(*A*)** and 31°C **(*B*)**. Light blue/light orange columns represent exposure to light and dark blue/dark orange columns exposure to dark. Error bars indicate representing mean ± SD (n = 3) values for three independent experiments. Statistical analysis was performed using t-tests for comparisons to the control ΔPc*ku70* strain (***P< 0.005, **P < 0.05, *P < 0.5).

At 27°C in the dark, conidiation was identical in all strains, while in the light we observed a slight reduction in conidiation with Δ*tom1* (**Figure 5A**). A more significant difference was observed at the elevated temperature of 31°C (**Figure 5B**). In light as well as darkness, conidiation was significantly reduced in Δ*tom1*, compared to the control strain. The wild-type phenotype in Δ*tom1::egfp-tom1* was not fully restored, which can be explained by ectopic integration of the wild-type gene in the recombinant transformants. In conclusion, *tom1* is involved in conidiation of *P. chrysogenum.*

## Discussion

Over the past few years, MAT-dependent gene expression has been investigated in several ascomycetous fungi such as *Aspergillus fumigatus*, *Fusarium graminearum*, *P. chrysogenum* and *Sordaria macrospora* (Klix et al., 2010; Becker et al., 2015; Kim et al., 2015; Yu et al., 2018; Ramsak et al., 2021). These analyses led to the identification of a wide variety of genes, many of which have not yet been functionally characterized. Among them is the *tom1* gene, encoding a 200 amino acid proline-rich protein with a predicted molecular mass of 21.7 kDa (**Supplementary Figure 3**). In a previous study, sequence comparison revealed that orthologous *tom1* genes are found exclusively in a monophyletic group of Eurotiomycetes (Ramsak et al., 2021).

In this study, we demonstrated that *tom1* is a functional protein, which is relevant for asexual development. Deletion of *tom1* resulted in reduced sporulation levels at elevated temperatures but did not affect colony diameters on plates. Expression of several other genes is directly regulated by MAT1-1-1 in *P. chrysogenum*, as was demonstrated for the *artA* gene involved in conidiospore germination (Becker et al., 2015). Thus it seems that MAT1-1-1 controls asexual development through several parallel pathways. It is known that asexual and sexual reproduction in euascomycetes are linked, for example by the *velvet* factor (Bayram and Braus, 2012). Since we did not investigate the function of *tom1* in sexual reproduction, some additional roles of *tom1* might remain undiscovered and require further research.

Although the *tom1* protein sequence has been thoroughly investigated, no known functional domain could be detected. However, we now show that *tom1* and its orthologs contain a short linear proline-proline-x–tyrosine motif, called PPxY, at the amino-terminus. This motif is known to be a ligand for small globular WW (Trp-Trp) domains, which are involved in diverse signalling pathways in eukaryotic organisms (Kay et al., 2000). In humans, the carboxyl-terminal domain (CTD) of mammalian RNA polymerase II contains a partial PY motif, which interacts with WW-domain containing TFs. Furthermore, these protein-protein interactions between PY motifs and TFs depend on the phosphorylation status of CTD (Gavva et al., 1997; Sudol and Hunter, 2000). A number of putative phosphorylation sites were predicted for *tom1*, and since the protein lacks any Pfam domains, we predict that it most likely interacts with other proteins to modulate downstream activities. It is thus possible that Tom1 interacts with the WW-domain containing TFs. However, further research is required to determine the precise function of the PPxY motif of Tom1.

Using *in vivo* reporter gene analysis, we confirmed that *tom1* is under the direct control of the α-domain MAT1-1-1 TF, which is consistent with previous studies (Becker et al., 2015; Ramsak et al., 2021). A significant enrichment of the MAT1-1-1 consensus binding motif has been observed adjacent to *tom1* TIS. It was described previously that the cognate TF binding site is surrounded by other TF binding sites that may also be degenerated (Zhang et al., 2006). Clustering of MAT1-1-1 and MAT1-2-1 motif binding sites within the promoters of their target genes has been noticed previously in *Neurospora crassa* and *P. chrysogenum* (Philley and Staben, 1994; Becker et al., 2015). Similarly, multiple clustered sequences have been found close to mating-related genes in *Schizosaccharomyces pombe* and *Ustilago maydis* (Bölker et al., 1992; Davey, 1992). Consensus binding sites of eukaryotic TFs are relatively short and dispersed throughout the genome. Accumulation of these short consensus sequences thus provides a favourable genomic landscape for functional target site selection, and help guide TFs to the target sites (Castellanos et al., 2020). Our *in vivo* reporter gene analysis suggests that the complete set of MAT1-1-1 binding sites is required for the proper expression of *tom1*. Thus, molecular aspects of these interactions need to be further investigated. However, we cannot exclude at this point that additional more general sequence motifs (e.g., for RNA polymerase II recruitment) are present downstream of the identified MAT1-1-1 binding site and that their absence in -149 and -222 prevents transcription.

We showed that *tom1* is likely under the control of the MAT1-1-1 and MAT1-2-1 heterodimer. Since fungal TFs represent only about 3-5% of protein-coding genes, it is common that they form cooperative associations with other regulators to control expression of a wide range of targets (Wortman et al., 2009; Carrillo et al., 2017). The consensus sequences for the HMG-domain MAT1-2-1 TF have been determined for *F. graminearum*, *N. crassa*, and *Podospora anserina* (Philley and Staben, 1994; Ait Benkhali et al., 2013; Kim et al., 2015). They all contain a central region comprising three thymine, flanked by either cytosine or guanine. Our analysis showed that the tom1-2 probe, which was used for EMSA experiments, does not contain a cluster of three thymines. However, it contains a motif which is recognized by HMG-domain SRY and diverse SOX (SRY-related HMG box) proteins (Mertin et al., 1999). The SRY TF is a key player in sex determination and SOX TFs play a regulatory role in a number of developmental processes in mammals, including humans (Vervoort et al., 2013). We speculate that the -AACAAT- sequence within the tom1-2 DNA probe may be also recognized by MAT1-2-1 from *P. chrysogenum*, a hypothesis that will be tested in our future analyses.

In *S. cerevisiae*, MAT1-1-1 orthologue MATα1 forms a complex with TF Mcm1, and together they activate expression of five sex-related genes (Galgoczy et al., 2004). In euascomycetes, interaction between α- and HMG-domain MAT TFs has been shown previously for the heterothallic fungus *N. crassa* (Badgett and Staben, 1999) and the homothallic (self-fertile) ascomycete *S. macrospora* (Jacobsen et al., 2002), although comparable interactions were not observed in other ascomycetes (Coppin et al., 1997; Zheng et al., 2013). Here, we demonstrate that MAT1-1-1 and MAT1-2-1 interact in the heterothallic *P. chrysogenum*. In addition, both MAT TFs are able to form homodimers. Cooperativity between TFs is an important mechanism through which they expand and diversify their expression-promoting patterns. Notably, ChIP-seq analysis identified 254 genes as being direct targets of MAT1-1-1 in *P. chrysogenum*. Therefore, we suggest that MAT1-1-1 acts in an assembly with MAT1-2-1 to mediate expression of such a diverse variety of genes. It is important to better understand the broad regulatory role of MAT1-1-1, and determine which genes are direct targets of MAT1-2-1.

Asexual sporulation is important strategy used by filamentous ascomycetes to quickly colonize and disperse in a favourable environment (Wyatt et al., 2013). Previously, functional characterization of *MAT1-1-1* revealed that its gene product is a positive regulator of sporulation in *Penicillium* species (Böhm et al., 2013; Mahmoudjanlou et al., 2020). In *Villosiclava virens*, deletion of *MAT1-1-1* resulted in abnormal conidial morphology (Yong et al., 2020). Overall, our findings add a new facet to the functionality of MAT1-1-1 regulators, further expanding our ideas about MAT-dependent pathways.

## Data Availability Statement

The original contributions presented in the study are included in the article/**Supplementary Material**. Further inquiries can be directed to the corresponding author.

## Author Contributions

BR: investigation, writing original draft, analysis and methodology. UK: supervision and methodology, conceptualization: Both authors took part in reviewing and editing of the manuscript.

## Funding

This study was funded by the German Research Foundation (DFG) (Bonn Bad-Godesberg, Germany) (KU 517/15-1). B.R. is a member of the Research Training Group GRK 2341 [Microbial Substrate Conversion (MiCon)]. We acknowledge support by the DFG Open Access Publication Funds of the Ruhr-Universität Bochum.

## Conflict of Interest

The authors declare that the research was conducted in the absence of any commercial or financial relationships that could be construed as a potential conflict of interest.

## Publisher’s Note

All claims expressed in this article are solely those of the authors and do not necessarily represent those of their affiliated organizations, or those of the publisher, the editors and the reviewers. Any product that may be evaluated in this article, or claim that may be made by its manufacturer, is not guaranteed or endorsed by the publisher.
