# Supplementary material for: The Penicillium chrysogenum tom1 Gene a Major Target of Transcription Factor MAT1-1-1 Encodes a Nuclear Protein Involved in Sporulation
Source: Front Fungal Biol. 2022 Jul 14;3:937023. doi: 10.3389/ffunb.2022.937023 (PMC10512297; doi:10.3389/ffunb.2022.937023)
Supplement: Supplementary file 1 [file DataSheet_1.docx]

**Supplementary Material**

**Supplementary Figure 1.** Generation and verification of Δtom1.

**Supplementary Figure 2.** 12% SDS-PAGE of purified heterogeneously produced GST-MAT1-1-1 and GST-MAT1-2-1 proteins.

**Supplementary Figure 3.** Schematic representation of the linear structure of Tom1.

**Supplementary Figure 4.** Flourescence microscopy.

**Supplementary Table 1.** Bacterial and fungal strains used in this study.

**Supplementary Table 2.** Oligonucleotides used in this study.

**Supplementary Table 3.** Plasmids used in this study.

**Supplementary Figures**

**Supplementary Figure 1**

**
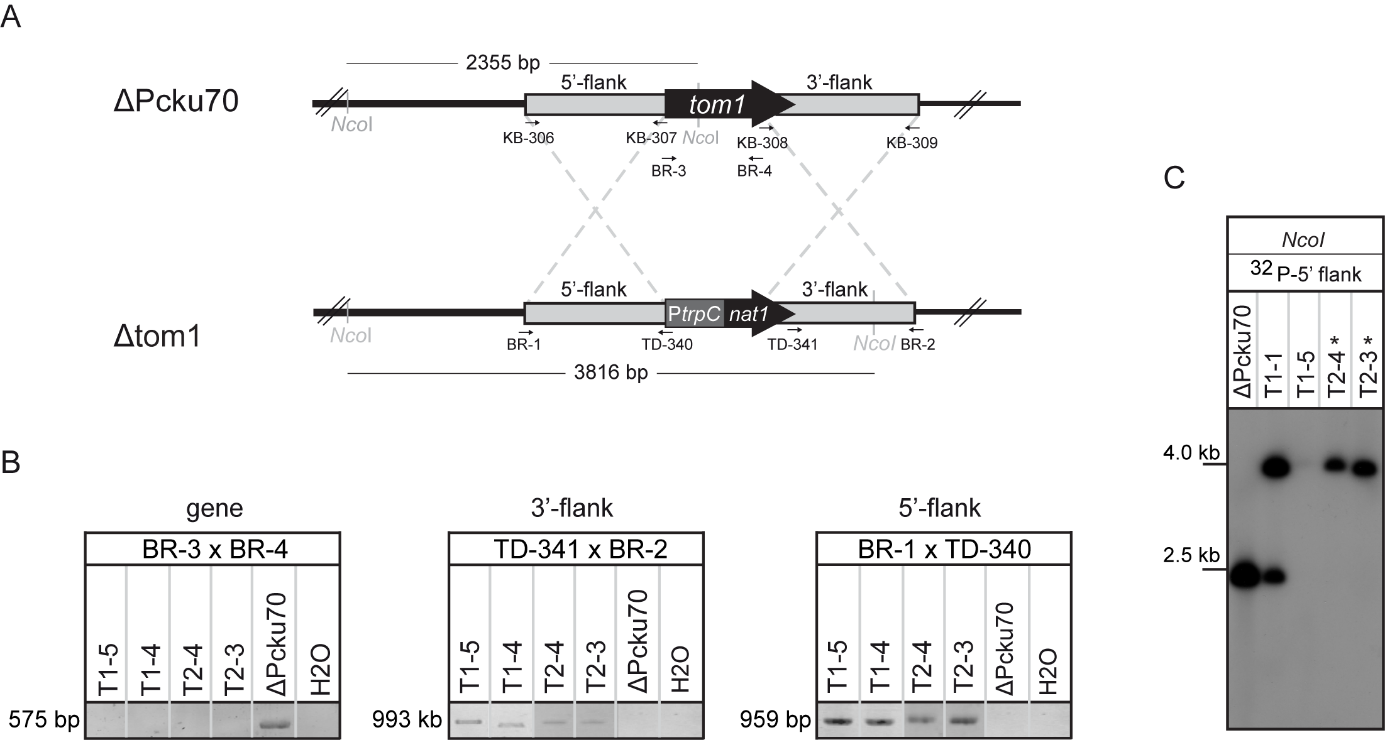
**

**Supplementary Figure 1.** Generation and verification of Δtom1. (A) Schematic illustration of the genomic situation of *tom1* locus in ΔPcku70 and Δtom1 strains. Gene replacement was reached by integration of a *nourseothricin* (*nat1*)-deletion cassette by homologous recombination. Positions of primers used for verification of successful integration of *nat1*-deletion cassette are marked with black arrows and corresponding names. Sizes of the expected signals (2355 bp for ΔPcku70 and 3816 bp for Δtom1 strain) of Southern hybridization are indicated. The restriction enzyme used for digestion of genomic DNA for Southern blot analysis are displayed in grey letters. Abbreviations: *nat1*, *nourseothricin*; P*trpC*, *Aspergillus nidulans* *trpC* promoter. (B) PCR analysis of the integration of the *nat1*-deletion cassette at the desired *tom1* gene locus in comparison to the ΔPcku70 strain. The positions of primers and corresponding fragments indicated in (A) could be detected. *tom1* deletion mutants are numbered T1-4, T1-5, T2-3, T2-4. The mutant T2-3 was used in our study. Genomic DNA from *P. chrysogenum* ΔPcku70 served as a control. H_2_O contains no genomic DNA. (C) Southern hybridization for verification of the Δtom1 strain (transformants T1-1, T1-5, T2-3, T2-4). Region spanning 5’ flank of the *tom1* gene was used as a probe for hybridization. successful integration of *nat1*-deletion cassette in transformants T2-3, T2-4 is indicated by asterisks. Genomic DNA of ΔPcku70 and Δtom1 strains was hydrolysed with *NcoI*.

**Supplementary Figure 2**


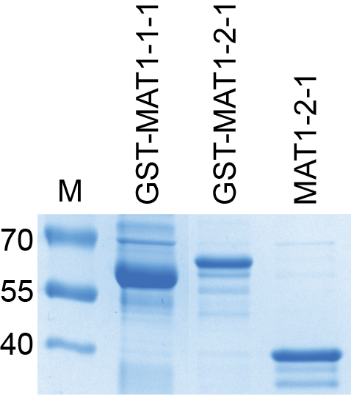


**Supplementary Figure 2.** 12% SDS-PAGE of purified heterogeneously produced GST-MAT1-1-1 and GST-MAT1-2-1 proteins. MAT1-2-1 was loaded on the gel, after removal of the GST-tag. The predicted molecular weights are: 64.8 kDa (GST-MAT1-1-1), 60 kDa (GST-MAT1-2-1) and 34.5 kDa (MAT1-2-1). Molecular marker (M) is indicated in kDa. Gel was stained with Coomassie blue stain.

**Supplementary Figure 3**


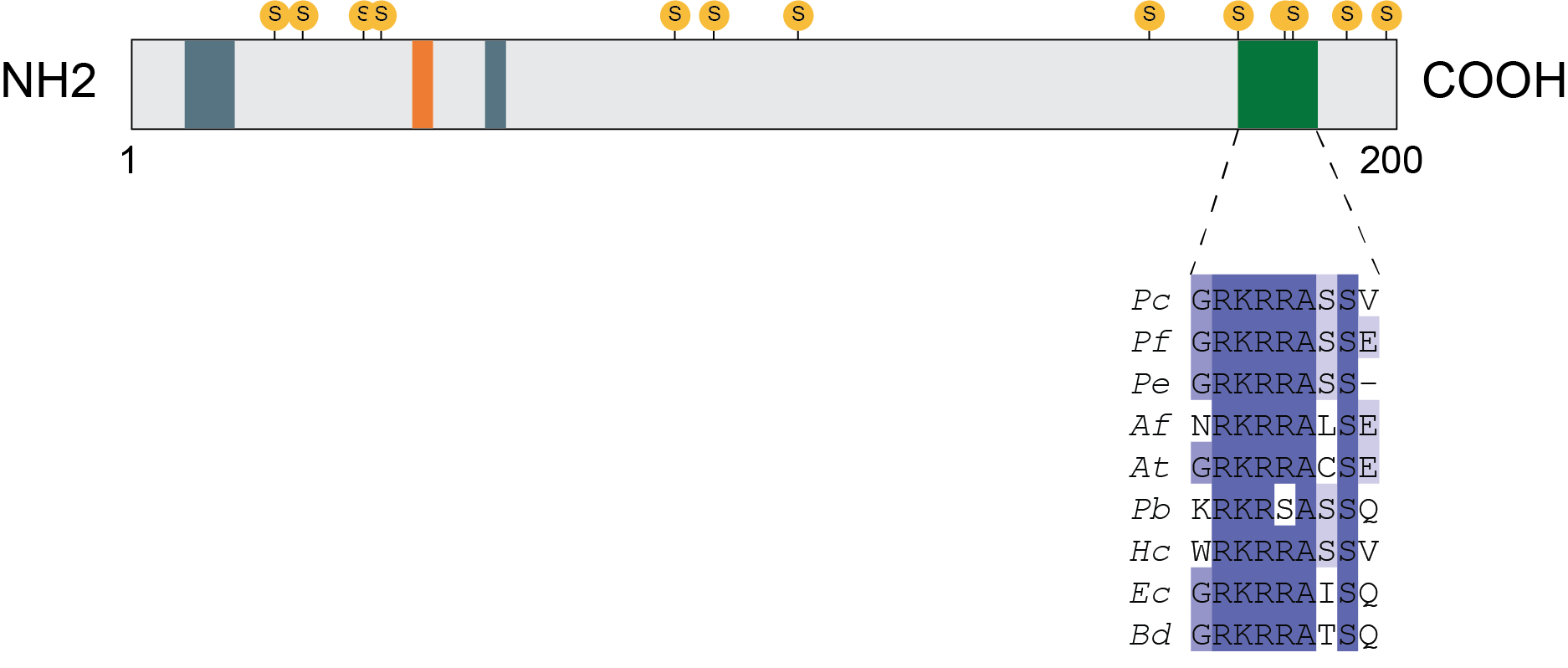


**Supplementary Figure 3.** Schematic representation of the linear structure of Tom1. The putative protein binding sites (blue), the linear PPxY (x = any amino acid) motif (orange) and putative phosphorylation sites (yellow) are indicated. The cut-value was set as 0.8. The conserved nuclear localization signals (NLS) is shown in green. This motif is conserved in other species belonging to Eurotiomycetes. Tom1 protein sequences from representative species *Pc*, *Penicillium chrysogenum*; *Pf*, *P. flavigenum*; *Pe*, *P. expansum*, *Af*, *Aspergillus fumigatus*; *At,* *A. terreus*; *Pb*, *Paracoccidioides brasiliensis*; *Hc*, *Histoplasma capsulatum*; *Ec*, *Emmonsia crescens*; *Bd*, *Blastomyces dermatitidis*. The putative protein binidng sites were predicted by online tool (<https://predictprotein.org/>). Putative phosphorylation residues were identified by NetPhos 3.1 server (Blom et al., 1999). The conserved nuclear localization signal (NLS) located at the C-terminus of Tom1 was identified previously (Ramsak et al., 2021).

**Supplementary Figure 4**


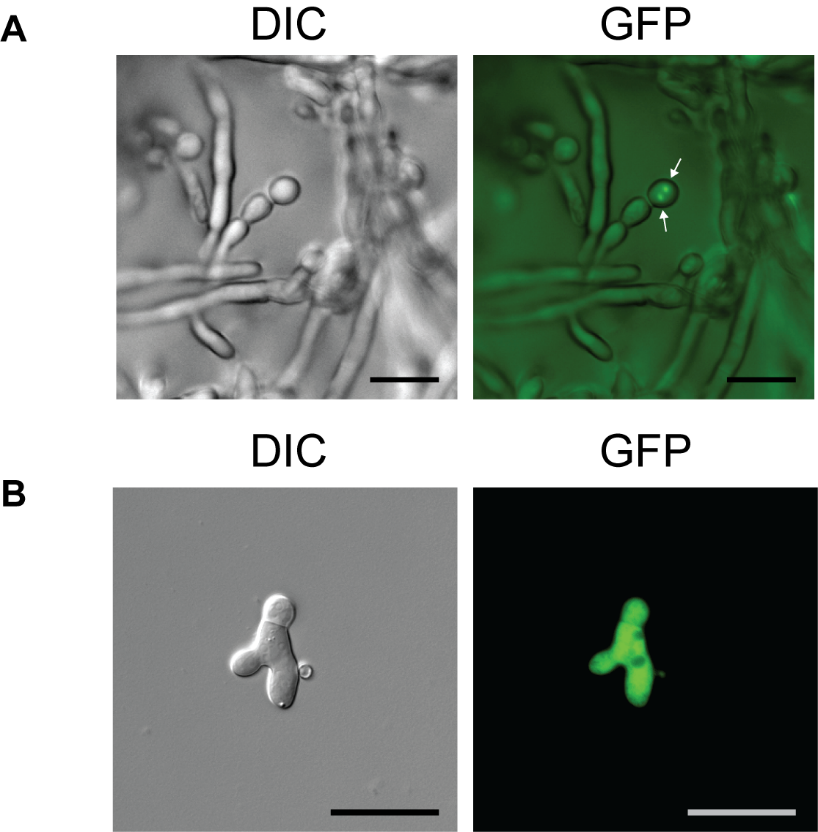


**Supplementary Figure 4.** Fluorescence microscopic localization of EGFP-Tom1 and EGFP. (A) In Δtom1::EGFP-Tom1 transformants tom1 localizes to the nucleus in conidia. Arrowheads indicate nuclei. (B) P2niaD18::EGFP transformants were used as a negative control. GFP fluorescence shows that enhanced green fluorescence protein (EGFP) is evenly distributed in the cytoplasm. Abbreviations: DIC, differential interference contrast; GFP, green fluorescence protein. Scale bars, 20 μm.

**Supplementary Tables**

**Supplementary Table 1.** Bacterial and fungal strains used in this study.

| **Strain** | **Genotype** | **Reference** |
| --- | --- | --- |
| ***E.coli*** | | |
| **XL1-Blue MRF’** | *recA1, endA1, gyrA96, thi1, hsdR17, supE44,relA1, lac-, [F’ proAB, laclqZΔM15, Tn10(tet^r^)]* | (Jerpseth et al., 1992) |
| **BL21-Gold (DE3)pLysS** | *E. coli* B F^–^ *ompT hsdS*(r_B_^–^ m_B_^–^) *dcm^+^* Tet^r^ *gal* λ(DE3) *endA* Hte [pLysS Cam^r^] | Agilent Technologies |
| ***S. cerevisiae*** |  |  |
| **PJ69-4a** | *MATa, TRP1-901, LEU2-3, 112, URA3-52, his3-200, gal4Δ, gal80Δ,GAL2UAS-GAL2TATA-ADE2, LYS2::GAL1UAS-GAL1TATA-HIS3, MET2::GAL7UAS GAL7TATA-lacZ* | (James et al., 1996) |
| **PJ69-4α** | *MATα, TRP1-901, LEU2-3, 112, URA3-52, his3-200, gal4Δ, gal80Δ,GAL2UAS-GAL2TATA-ADE2, LYS2::GAL1UAS-GAL1TATA-HIS3, MET2::GAL7UAS GAL7TATA-lacZ* | (James et al., 1996) |
| ***P. chrysogenum*** | | |
| **P2niaD18** | Penicillin producer, *niaD^−^* (*MAT1-1-1* idiomorph) | (Hoff et al., 2008) |
| **Δ*Pcku70*** | P2niaD18; *ΔPcku70*::*FRT; niaD^−^* | (Kopke et al., 2010) |
| **PC3** | Wild type (*MAT1-2-1* idiomorph) | (Böhm et al., 2013) |
| **MAT1-ChIP** | P2niaD18; P*gpd*::*EGFP*::*MAT1-1-1*::T*trpC; nat1; niaD^−^* | (Becker et al., 2015) |
| **P2niaD18*::EGFP*** | P2niaD18; P*gpd*::*EGFP*::T*trpC;* *ble; niaD^−^* | this study |
| **P2niaD18*::EGFP-tom1*** | P2niaD18; P*gpd*::*EGFP-tom1*::T*trpC;* *ble; niaD^−^* | this study |
| **Δ*tom1*** | *ΔPcku70*; *Δtom1::nat1*::T*trpC; niaD^−^* | this study |
| **Δ*tom1::EGFP-tom1*** | *ΔPcku70*; *Δtom1*::*nat1*, P*gpd*::*EGFP-tom1*, *ble; niaD^−^* | this study |
| **P2niaD18 + P*tom1::DsRed*** | P2niaD18; P*tom1*::*DsRed*::T*trpC*; *ble*; *niaD^−^* | this study |
| **EGFP-MAT1 + P*tom1::DsRed*** | P2niaD18; P*tom1*::*DsRed*::T*trpC*; P*gpd*::*EGFP*::*MAT1-1-1*::T*trpC; nat1; ble*; *niaD^−^* | this study |
| **EGFP-MAT1 + P*tom1*_(-149_*_)_::DsRed*** | P2niaD18; P*tom1_(-149-965)_*::*DsRed*::T*trpC*; P*gpd*::*EGFP*::*MAT1-1-1*::T*trpC; nat1; ble*; *niaD^−^* | this study |
| **EGFP-MAT1 + P*tom1* _(-222)_*::DsRed*** | P2niaD18; P*tom1_(-222-965)_::DsRed::*T_trpC_; *P_gpd_::EGFP::MAT1-1-1::T_trpC_; nat1; ble*; *niaD^−^* | this study |

**Supplementary Table 2.** Oligonucleotides used in this study.

| **Oligonucleotide** | **Sequence (5’-3’)** |
| --- | --- |
| BR-1 | GGAGTTGCAAGGAGTCGATATTGCTC |
| BR-2 | CCTAACTTTGCCCAAGCAAGGTGGAT |
| BR-3 | GGCAAGTCGCAATACTTCTT |
| BR-4 | CCACAGGCTCCTGCATTAAC |
| PtrpC_seq_r (TD-340) | CTCCACTAGCTCCAGCCAAG |
| nat1_seq_f (TD-341) | GGCGCTCTACATGAGCATG |
| 5‘-Flanke Pc20g00090_IF_f (KB-306) | CGGTACCACGCATGCTGCAGCGAGATGCAGATGAACTTGC |
| 5‘-Flanke Pc20g00090_IF_r (KB-307) | CACGAATTCTGGATCCGTGATATGACCAGAGTGAAG |
| 3‘-Flanke Pc20g00090_IF_f (KB-308) | ATTCGTCGACAAGCTTCTATGAGCTGTTCCGTGAG |
| 3‘-Flanke Pc20g00090_IF_r (KB-309) | TAGAATACAGCGGCCGCCGATCCACCATATCACATGC |
| tom1_KpnI_f | CTCAGGTACCATGATGCCTCCATCAGAC |
| tom1_EcoRI_r | CGCAGAATTCGGATTCATGAGACCAGGG |
| TtrpC reverse | AGCTGACATCGACACCAACG |
| BspHI-MAT1-2-1_f | TATATTCATGATGGCGAAAACCCTCTTG |
| MAT1-2-1-EcoRI_r | ATATAGAATTCTTAGAACACGCTGTTCATAGGG |
| **EMSA** |  |
| tom1-2 | TCACGTGATCTCTATTGAGAACAATAGAA |
| kex2-3 | GCTTATTCAAACAGAAGCTAATTCCTTTG |
| ***In vivo* *dsRed* expression study** | |
| Pc20g00090_dsRed_ApaI_f | ATAGGGCCCCGAGATGCAGATGAACTT |
| Pc20g00090_dsRed_HindIII | CGACGAAGCTTGTGATATGACCAGAGTGA |
| Pc20g00090_753_ds Red_HindIII | CCGAAGCTTGCTATTGTTCATGGGG |
| Pc20g00090_826_ds Red_HindIII | ACGTAAGCTTCGTTACGGGCCATTGAC |
| **Yeast two-hybrid analysis** | |
| mat1-2_for_NdeI | ACTGCATATGGGACTTGACCTCGGTGATC |
| MAT1-2-1_Nt_rev | ATAACATATGATGATGGCGAAAACCCTCTTG |
| MAT-1-2-1-BD_rev_EcoRI | GATTGAATTCCTAAGAAGGGCGACGAGG |
| mat1-2_rev_EcoRI | TTGAGAATTCTTAGAACACGCTGTTCATAG |
| mat1-1-250_rev_STOP | TCACTCGAGCTAGTTGTTATCGATGCAGTGC |
| mat1-1-109_for | GCTAAGAATTCATGCAGAATGTACTGTCCTCTCCT |
| mat1-1_rev | CAGACTCGAGCTAGTTGTGCCCAAAGATCC |

**Supplementary Table 3.** Plasmids used in this study.

| **Plasmid** | **Characteristics** | **Reference/Source** |
| --- | --- | --- |
| pD-NAT1 | P*trpC* promoter from *A. nidulans*, *nat1* resistance gene of *Streptomyces noursei;* used for construction of KO-EN45_061320 | (Kück and Hoff, 2006) |
| KO-EN45_061320 | Deletion plasmid for *tom1* (*EN45_061320*) 5’ flank *tom1*, *trpC(p)::nat1*, 3’ flank, *tom1* ura3, *bla* | this study |
| Yas1_ptrpC-phleo-egfp | P*gpd* of *A. nidulans*, *egfp*, T*trpC* of *A. nidulans*, *ble* resistance gene of *Streptoalluteichus hindustanus;* used for construction of pGFP-tom1_ble | Mahmoudjanlou  (Unpublished data) |
| pGFP-Tom1_ble | P*gpd* of *A. nidulans*, *egfp*, *tom1* gene of *P. chrysogenum*, T*trpC* of *A. nidulans*, *ble* resistance gene of *Streptoalluteichus hindustanus;* used for localization and complementation studies | this study |
| ***In vivo* *dsRed* expression study** | | |
| pDsRed | *dsRed* gene of *Discosoma* sp., *TtrpC* of *A. nidulans* | (Becker et al., 2015) |
| pDsRed_Ptom1 | P*_tom1_* _(0-1000)_ of *P. chrysogenum*, *dsRed* gene of *Discosoma sp.*, T*trpC* of *A. nidulans*, *ble* resistance gene of *Streptoalluteichus hindustanus* | this study |
| pDsRed_Ptom1(-149) | P*_tom1_*_(-149-1000)_ of *P. chrysogenum*, *dsRed* gene of *Discosoma sp.*, T*trpC* of *A. nidulans*, *ble* resistance gene of *Streptoalluteichus hindustanus* | this study |
| pDsRed_Ptom1(-222) | P*_tom1_*_(-222-1000)_ of *P. chrysogenum*, *dsRed* gene of *Discosoma sp.*, T*trpC* of *A. nidulans*, *ble* resistance gene of *Streptoalluteichus hindustanus* | this study |
| **Protein expression** | | |
| pGEX-MAT1-1-1 | *MAT1-1-1* cDNA sequence of *P. chrysogenum*; used for heterologous expression of a GST-MAT1-1-1 | (Becker et al., 2015) |
| pGEX-MAT1-2-1 | *MAT1-2-1* cDNA sequence of *P. chrysogenum*; used for heterologous expression of a GST-MAT1-2-1 | this study |
| **Yeast Two-hybrid analysis** | | |
| pGADT7 | bait vector for yeast one-hybrid analysis,  *S. cerevisiae PADH1 and TADH1, gal4-AD* (aa 768-881)*, LEU2, amp^r^* | Clontech |
| pGBKT7 | pray vector for yeast one-hybrid analysis,  *S. cerevisiae PADH1 and TADH1, gal4-BD* (aa 1-147)*, TRP1, kan^r^* | Clontech |
| pA-RanBPM | *ranbpm* cDNA (aa 51-654) in pGADT7, *LEU2*, *amp^r^* | (Tucker et al., 2009) |
| pA-MAT1-NtBD | *MAT1-1-1* cDNA (aa 1-220) in pGADT7, *LEU2*, *amp^r^* | this study |
| pA-MAT1-CtBD | *MAT1-1-1* cDNA (aa 109-342) in pGADT7, *LEU2*, *amp^r^* | this study |
| pA-MAT2 | *MAT1-2-1* cDNA (aa 1-303) in pGADT7, *LEU2*, *amp^r^* | this study |
| pA-MAT2-Nt | *MAT1-2-1* cDNA (aa 1-131) in pGADT7, *LEU2*, *amp^r^* | this study |
| pB-MAT1-Nt | *MAT1-1-1* (aa 1-118) in pGBKT7, *TRP1*, *kan^r^* | Wolfers  (unpublished data) |
| pB-MAT1-NtBD | *MAT1-1-1* (aa 1-220) in pGBKT7, *TRP1*, *kan^r^* | this study |
| pB-MAT2-Nt | *MAT1-2-1* (aa 1-131) in pGBKT7, *TRP1*, *kan^r^* | this study |
| pB-MAT2-NtBD | *MAT1-2-1* (aa 1-207) in pGBKT7, *TRP1*, *kan^r^* | this study |

**References**

Becker, K., Beer, C., Freitag, M., and Kück, U. (2015). Genome-wide identification of target genes of a mating-type α-domain transcription factor reveals functions beyond sexual development. Mol Microbiol 96: 1002–1022.

Böhm, J., Hoff, B., O’Gorman, C. M., Wolfers, S., Klix, V., Binger, D., et al. (2013). Sexual reproduction and mating-type–mediated strain development in the penicillin-producing fungus *Penicillium chrysogenum*. Proc Natl Acad Sci U S A 110: 1476–1481.

Hoff, B., Pöggeler, S., and Kück, U. (2008). Eighty years after its discovery, Fleming’s *Penicillium* strain discloses the secret of its sex. Eukaryot Cell 7: 465–470.

James, P., Halladay, J., and Craig, E. A. (1996). Genomic libraries and a host strain designed for highly efficient two-hybrid selection in yeast. Genetics 144: 1425–1436.

Jerpseth, B., Greener, A., Short, J., Viola, J., and Kretz, P. (1992). XL1-blue MRF’ *E. coli* cells: McrA-, McrCB-, mcrF-, Mmr-, HsdR- derivative of XL1-blue cells. Strategies 5, 81–83.

Kopke, K., Hoff, B., and Kück, U. (2010). Application of the *Saccharomyces cerevisiae* FLP/FRT recombination system in filamentous fungi for marker recycling and construction of knockout strains devoid of heterologous genes. Appl Environ Microbiol 76: 4664–4674.

Kück, U., Hoff, B. (2006). Application of the nourseothricin acetyltransferase gene (*nat1*) as dominant marker for the transformation of filamentous fungi. Fungal Genet Newsl 53: 9–11.

Tucker, C. L., Peteya, L. A., Pittman, A. M. C., and Jing, Z. (2009). A genetic test for yeast two-hybrid bait competency using RanBPM. Genetics 182: 1377–1379.
